# Structural Definition of Duck Major Histocompatibility Complex Class I Molecules That Might Explain Efficient Cytotoxic T Lymphocyte Immunity to Influenza A Virus

**DOI:** 10.1128/JVI.02511-16

**Published:** 2017-06-26

**Authors:** Yanan Wu, Junya Wang, Shuhua Fan, Rong Chen, Yanjie Liu, Jianhua Zhang, Hongyu Yuan, Ruiying Liang, Nianzhi Zhang, Chun Xia

**Affiliations:** a Department of Microbiology and Immunology, College of Veterinary Medicine, China Agricultural University, Beijing, China; b Key Laboratory for Insect-Pollinator Biology of the Ministry of Agriculture, Institute of Apiculture, Chinese Academy of Agricultural Sciences, Beijing, China; c Key Laboratory of Animal Epidemiology and Zoonosis, Ministry of Agriculture, China Agricultural University, Beijing, China; Icahn School of Medicine at Mount Sinai

**Keywords:** crystal structure, duck, MHC class I, influenza A virus, disulfide bond

## Abstract

A single dominantly expressed allele of major histocompatibility complex class I (MHC I) may be responsible for the duck's high tolerance to highly pathogenic influenza A virus (HP-IAV) compared to the chicken's lower tolerance. In this study, the crystal structures of duck MHC I (*Anpl*-UAA*01) and duck β2-microglobulin (β2m) with two peptides from the H5N1 strains were determined. Two remarkable features were found to distinguish the *Anpl*-UAA*01 complex from other known MHC I structures. A disulfide bond formed by Cys^95^ and Cys^112^ and connecting the β5 and β6 sheets at the bottom of peptide binding groove (PBG) in *Anpl*-UAA*01 complex, which can enhance IAV peptide binding, was identified. Moreover, the interface area between duck MHC I and β2m was found to be larger than in other species. In addition, the two IAV peptides that display distinctive conformations in the PBG, B, and F pockets act as the primary anchor sites. Thirty-one IAV peptides were used to verify the peptide binding motif of *Anpl*-UAA*01, and the results confirmed that the peptide binding motif is similar to that of HLA-A*0201. Based on this motif, approximately 600 peptides from the IAV strains were partially verified as the candidate epitope peptides for *Anpl*-UAA*01, which is a far greater number than those for chicken BF2*2101 and BF2*0401 molecules. Extensive IAV peptide binding should allow for ducks with this *Anpl*-UAA*01 haplotype to resist IAV infection.

**IMPORTANCE** Ducks are natural reservoirs of influenza A virus (IAV) and are more resistant to the IAV than chickens. Both ducks and chickens express only one dominant MHC I locus providing resistance to the virus. To investigate how MHC I provides IAV resistance, crystal structures of the dominantly expressed duck MHC class I (p*Anpl*-UAA*01) with two IAV peptides were determined. A disulfide bond was identified in the peptide binding groove that can facilitate *Anpl*-UAA*01 binding to IAV peptides. *Anpl*-UAA*01 has a much wider recognition spectrum of IAV epitope peptides than do chickens. The IAV peptides bound by *Anpl*-UAA*01 display distinctive conformations that can help induce an extensive cytotoxic T lymphocyte (CTL) response. In addition, the interface area between the duck MHC I and β2m is larger than in other species. These results indicate that HP-IAV resistance in ducks is due to extensive CTL responses induced by MHC I.

## INTRODUCTION

Influenza A virus (IAV) poses a large threat for both animal and human health and is a growing problem (1–4). Ducks play a pivotal role in its epidemiology because they are natural reservoirs of IAV (5). Although all subtypes of IAV are perpetuated in ducks (6, 7), they typically do not show serious signs of the disease, even that caused by highly pathogenic IAVs (HP-IAVs), such as the H5N1 strain, which is lethal to chickens (8, 9). Since 2002, many Asian lineage H5N1 HP-IAVs have been shown to produce similar symptoms and mortality, although clinical outcomes are also affected by the age and species of the ducks and the strains of IAVs (10, 11). Moreover, ducks have more active and robust cellular immune responses than chickens (12). These findings suggest that at least some species of duck show efficient immune responses against IAV infection.

Studies have indicated that major histocompatibility complex class I (MHC I) plays a role in the anti-IAV response (13–15). MHC I can present viral epitope peptides to specific T cell receptors (TCRs), resulting in the proliferation of cytotoxic T lymphocytes (CTLs) and eventual clearance of the virus from the host (16–18). CTL responses mediated by MHC I play a significant role in primary IAV infection and provide cross-protection against different IAV strains in chickens, mice, and humans (19–23). Suppressive subtractive hybridization libraries have been constructed to enrich differentially expressed genes from the lungs of ducks infected with IAVs of high or low pathogenicity compared to mock-infected controls (13). These data showed that the MHC I gene was upregulated in ducks under both conditions, especially in the highly pathogenic group, in which the MHC I gene was increased by more than 1,000-fold (13). Another study showed that there was an increase in MHC I gene expression in two different duck species after injection with a commercial inactivated vaccine (24). These results suggest that duck MHC I may play a role similar to that of the human HLA against infection.

MHC I molecules are encoded by polymorphic alleles from several loci. These loci make up the peptide binding groove (PBG) and contain epitope peptides against multiple pathogens. In humans and mice, there are three functional MHC I loci that provide polymorphic alleles for peptides (25, 26); however, many birds predominantly express only one MHC class I gene, such as in chickens and ducks (27). Chickens express only one dominant MHC I locus, referred to as BF2, which is adjacent to the *TAP* gene (28). Although there are five different loci in the duck MHC I genome region (named the UAA to UEA loci), only UAA, which lies adjacent to the polymorphic *TAP2* gene, is predominantly expressed for the CTL immune response (29, 30). To date, more than 400 structures of the peptide-MHC I-β2-microglobulin β2m) protein complexes (here called pMHC I) of different species have been determined, and most of them are from human and mouse. Structural studies revealed that the epitope peptides are fixed in the PBG of the MHC I heavy chain by six pockets (A to F) (31). Some pockets, typically the B and F pockets, play a critical role to bind the peptides and determine the peptide binding motif of a certain MHC I molecule. In recent years, pMHC I structures of other species have been solved, while in avian species, only chicken MHC I (BF2) was resolved (32–34). These structures have greatly facilitated the identification of MHC I-restricted CTL epitopes.

Several studies have indicated that “minimal MHC” is related to resistance and the susceptibility of chickens to viruses, such as the Rous sarcoma virus and Marek's disease virus (MDV) (32, 35, 36). Chickens expressing BF2*0401 (B4 haplotype) are susceptible to MDV, and chickens expressing BF2*2101 (B21 haplotype) are resistant to MDV. Crystal studies of BF2 structures have illustrated its resistance or susceptibility to MDV (32). The MHC I complex presents viral peptides to CTLs, depending on the six (A to F) pockets in its peptide binding groove (PBG). The MHC I polymorphisms determine the distinct three-dimensional (3D) structure of the MHC I PBG, and each classical MHC I molecule has a specific peptide binding motif. Crystal studies of B21 have shown that it can remodel its pockets to accommodate different peptides based on its wide binding groove and interplay of two charged residues (Arg^9^ and Asp^24^); B21 could bind multiple peptides from MDV and activate extensive T cell repertories to clear MDV (32). In contrast, B4 has a narrow peptide binding groove and strong polar pockets that restrict its peptide binding motif and the presented MDV peptides (33). Therefore, the CTL response to MDV induced by B4 is not strong enough to prevent infection and suggests that resistance is controlled by the type of MHC I and its 3D structure.

MHC I could also play a role in the susceptibility of viruses. For IAV, the higher resistances of some species of duck indicate that their MHC I alleles would present more IAV peptide epitopes than those of chickens (11, 14). Our previous data, as well as a recently published study, confirmed the presence of two or more cysteines, which have never been found in humans or other species, in the PBGs of many duck MHC I alleles (30, 37). To date, little is known about the 3D structure and peptide presentation of duck MHC I. In this study, we determined two crystal structures of the dominantly expressed duck MHC I (*Anpl*-UAA*01) molecules with two IAV peptides. The crystal structures showed that *Anpl*-UAA*01 exhibits characteristics consistent with the duck MHC I-peptide-β2m complex (pMHC I) architecture; however, we also identified a disulfide bond in its PBG. Unlike the chicken MHC I, only B and F pockets of *Anpl*-UAA*01 are the primary anchor sites for peptide binding. The two pockets, whose compositions are similar to those of the B and F pockets of HLA-A2, could accommodate diverse residues and make *Anpl*-UAA*01 an extensive peptide binding motif. By screening the entire sequences of IAV strains, approximately 600 nonapeptides fit the identified peptide binding motif of *Anpl*-UAA*01. Our study demonstrates that *Anpl*-UAA*01 could present more IAV epitope peptides, suggesting that ducks with this MHC I haplotype may be protected from infection.

## RESULTS

### The *Anpl*-UAA*01 structure shows an unexposed disulfide bond in the PBG and a large interface between heavy and light (H and L) chains.

**FIG 1:**
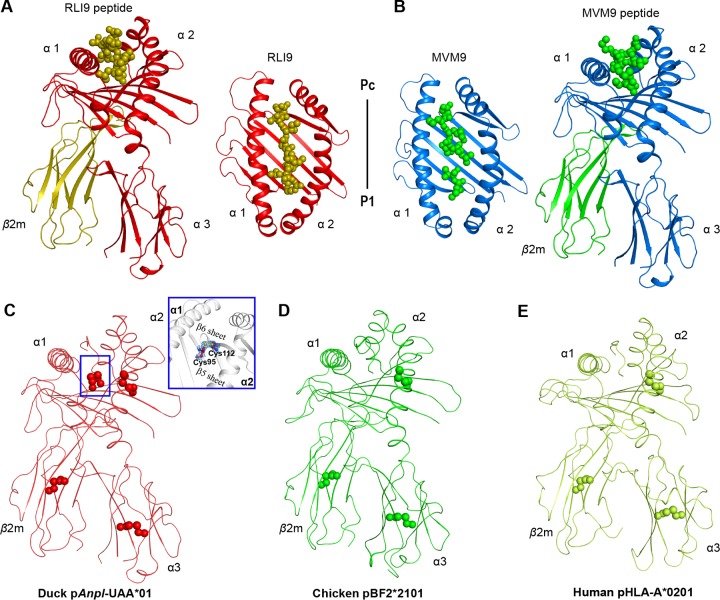
Structural overview and additional disulfide bond of the p*Anpl*-UAA*01 complex. (A and B) The overall structures of p*Anpl*-UAA*01–RLI9 (A) and p*Anpl*-UAA*01–MVM9 (B). p*Anpl*-UAA*01 chains are shown with distinct colors: for p*Anpl*-UAA*01–RLI9, H in red and L in olive, and for p*Anpl*-UAA*01–MVM9, H in marine and L in green). Peptides are shown as spheres using the same colors with their light chains. (C to E) The additional disulfide bond of p*Anpl*-UAA*01 compared with chicken and human pMHC I complexes. Disulfide bonds are marked with spheres. The additional disulfide bond formed by Cys^95^-Cys^112^ of p*Anpl*-UAA*01 is labeled in a box and amplified to show its position and electronic density map. Both chicken pBF2*2101 (green, Protein Data Bank code 3BEV) and human pHLA-A*0201 (lemon, Protein Data Bank code 3PWN) have just three disulfide bonds distributed in the α2 and α3 domains and β2m chains.

**FIG 2:**
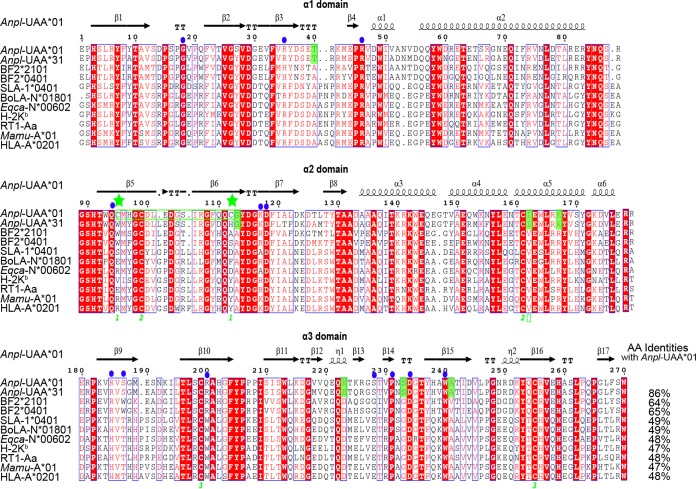
Structure-based amino acid sequence alignment of *Anpl*-UAA*01 and representatives of other crystallized MHC I molecules, with the secondary structure elements indicated. Black arrows above the alignment indicate β-strands; cylinders denote α-helices. Green numbers denote residues that form disulfide bonds. Cysteines at positions 95 and 112 are marked by green stars. Conserved residues are highlighted in red. Residues highlighted in green are species-specific amino acids that differ between duck and other animals. Solid blue dots indicate that the residues interact with *Anpl*-β2m. The total amino acid (AA) identities between *Anpl*-UAA*01 and the listed MHC I molecules are given at end of each sequence.

Two trimer complexes formed by *Anpl*-UAA*01, duck β2m (*Anpl*-β2m), and the MVM9 and RLI9 peptides were crystallized in the P1211 space group at resolutions of 1.71 Å and 2.06 Å, respectively (Table 1). Both of the complexes displayed a canonical pMHC I structure, in which the peptide was located in the platform formed by the α1 and α2 domains of *Anpl*-UAA*01 above the α3 domain and light-chain *Anpl*-β2m (Fig. 1A and B). The most remarkable features of the p*Anpl*-UAA*01 complexes are the two disulfide bonds formed by 4 cysteines in the PBG (Fig. 1C). In other known pMHC I structures, there is only one disulfide bond formed by a pair of cysteines in their PBGs, such as in chicken pBF2*2101 (32) and human pHLA-A*0201 (38) (Fig. 1D and E). These two cysteines have been found only in ducks (Fig. 2). This novel disulfide formed by Cys^95^ and Cys^112^ of p*Anpl*-UAA*01 complexes connects the β5 and β6 sheets at the bottom of the PBG.

**TABLE 1:** Data collection and refinement statistics for p*Anpl*-UAA*01–MVM9/RLI9

Parameter	Value^*a*^ for:	
	p*Anpl*-UAA*01–MVM9	p*Anpl*-UAA*01–RLI9
Data collection		
Space group	P1211	P1211
Cell dimensions		
*a*, *b*, *c* (Å)	46.27, 64.39, 77.92	46.49, 63.54, 79.95
α, β, γ (°)	90.00, 105.74, 90,00	90.00, 106.10, 90.00
Resolution (Å)	50.00–1.80 (1.86–1.80)	50.00–2.05 (2.09–2.05)
No. of reflections		
Total	112,298	180,543
Unique	41,752	27,433
*R*_sym_ or *R*_merge_^*b*^	0.063 (0.542)	0.092 (0.568)
*I*/σ*I*	20.759 (2.667)	24.100 (3.691)
Completeness (%)	96.2 (98.2)	99.8 (99.9)
Redundancy	4.5 (4.7)	4.1 (4.1)
Refinement		
Resolution (Å)	50.00–1.71	50.00–2.06
No. of reflections	40612	26122
*R*_work_/*R*_free_ (%)^*c*^	18.27/21.06	20.80/25.33
Root mean square deviation		
Bond length (Å)	0.019	0.006
Bond angle (°)	1.931	1.057
Avg B factor	32.794	33.678
Ramachandran plot quality (%)		
Most favored region	98.33	98.92
Allowed region	1.67	1.08
Disallowed	0.00	0.00

a Values in parentheses are for the highest-resolution shell.

b *R*_merge_ = Σ*_hkl_* Σ*_i_* |*I_i_*(*hkl*) − 〈*I*(*hkl*)〉 |/Σ*_hkl_* Σ*_i_ I_i_*(*hkl*), where *I_i_*(*hkl*) is the observed intensity and 〈*I*(*hkl*)〉 is the average intensity from multiple measurements.

c *R* = Σ*_hkl_* || *F*_obs_ | − *k* | *F*calc | |Σ*_hkl_* | *F_obs_* |, where *R*_free_ is calculated for a randomly chosen 5% of reflections and *R*_work_ is calculated for the remaining 95% of reflections used for structure refinement.

**FIG 3:**
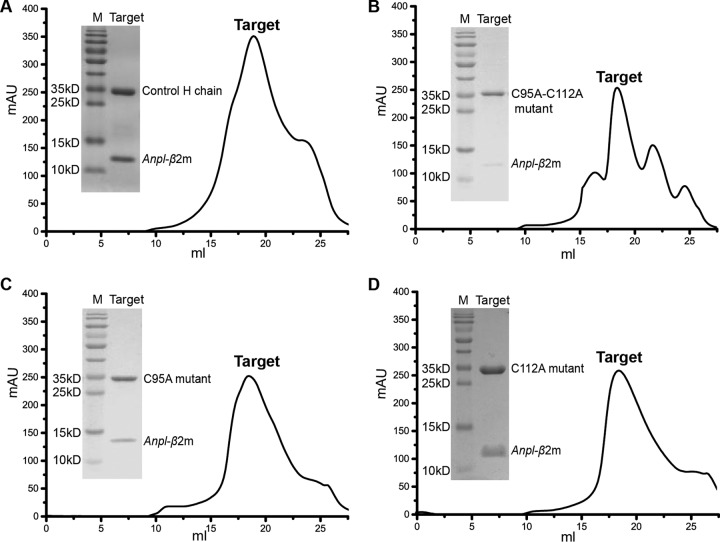
Gel filtration and SDS-PAGE results of no-peptide refolding. *Anpl*-UAA*01 and *Anpl*-β2m can co-refold in the absence of participation of the peptide, either the wild-type *Anpl*-UAA*01 heavy chain or the C-A mutant. Different heavy chains and *Anpl*-β2m are marked in the corresponding positions. Target, co-refolding products eluted via gel filtration. M, protein ladder. (A) Co-refolding results for the wild-type *Anpl*-UAA*01 and *Anpl*-β2m. (B) Co-refolding results for the C95A-C112A double mutant heavy chain and *Anpl*-β2m. (C) Co-refolding results for the C95A mutant heavy chain and *Anpl*-β2m. (D) Co-refolding results for the C112A mutant heavy chain and *Anpl*-β2m.

The no-peptide refolding product (only *Anpl*-UAA*01 and *Anpl*-β2m) was used as a negative control to judge the refolding efficiencies of different peptides; however, a peak (approximately 300 milli-absorbance units [mAU]) eluted at the position representing the p*Anpl*-UAA*01. The gel filtration and SDS-PAGE results showed that the peak represented the complex of *Anpl*-UAA*01 and *Anpl*-β2m (Fig. 3), which is different from results of studies on cattle, pig, chicken, and horse pMHC I refolding (33, 39–41). This result could be due to the Cys^95^-Cys^112^ disulfide bond of p*Anpl*-UAA*01. In order to clarify the impact of this disulfide bond, Cys^95^ and Cys^112^ were mutated to alanine (A), alone and together. However, the results for refolding of the mutated *Anpl*-UAA*01s were similar to those for the wild type (Fig. 3), indicating that the additional disulfide bond is not directly related to the stability of *Anpl*-UAA*01 without peptide binding.

**FIG 4:**
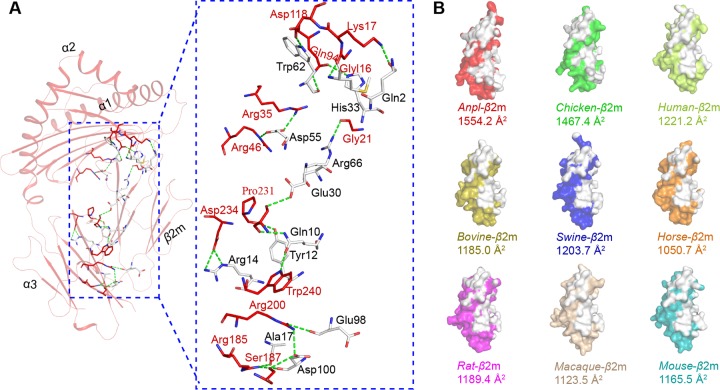
Strong binding between duck H and L chains. (A) Interactions between H and L chains of p*Anpl*-UAA*01. p*Anpl*-UAA*01 is shown in red, with the interactive residues shown as sticks and colored according to the atom type (blue, N; red, O). The interactive residues on the L chain are colored white. The hydrogen bonds and salt bridges are shown as green dashed lines. All of the interactions and interactive residues are magnified within a blue dotted box and labeled. (B) Interface areas of the L chains of p*Anpl*-UAA*01 and pMHC I structures from the other known species. All the L chains are surface representations with different colors. The white areas indicate the interactive regions contacting H chains. Detailed interface areas are shown under each structure. The structures are as follows: chicken (BF2*2101, PDB code 3BEV), human (HLA-A2, PDB code 3PWN), bovine (BoLA-N*01801, PDB code 3PWU), swine (SLA-1*0401, PDB code 3QQ3), horse (Eqca-N*00602, PDB code 4ZUV), rat (RT1-Aa, PDB code 1ED3), macaque (Mamu-A*01, PDB code 1ZVS), and mouse (H-2 Kb, PDB code 3TID).

The formation of a complex without the peptide may be due to the strong binding affinity between H and L chains. There are a total of 18 hydrogen bonds and salt bridges between *Anpl*-UAA*01 and *Anpl*-β2m (Fig. 4A), which is greater than the numbers found in the pMHC I structures of chickens (13) (pBF2*0401, PDB code 4E0R) and pigs (16) (pSLA-1*0401, PDB code 3QQ3). In addition, the size of the interface between *Anpl*-UAA*01 and *Anpl*-β2m is 1,554.2 Å^2^, which is the largest among the known pMHC I structures of different species (Fig. 4B), including human (PDB code 3PWN), monkey (PDB code 1ZVS), mouse (PDB code 3TID), rat (PDB code 1ED3), swine (PDB code 3QQ3), cattle (PDB code 3PWU), horse (PDB code 4ZUV), and chicken (PDB code 3BEV) (33, 38–44). The interactions and interface area between *Anpl*-UAA*01 and *Anpl*-β2m likely contribute to the stability of the complex without binding peptides.

### The distinct peptide conformations presented by the *Anpl*-UAA*01 molecule.

**FIG 5:**
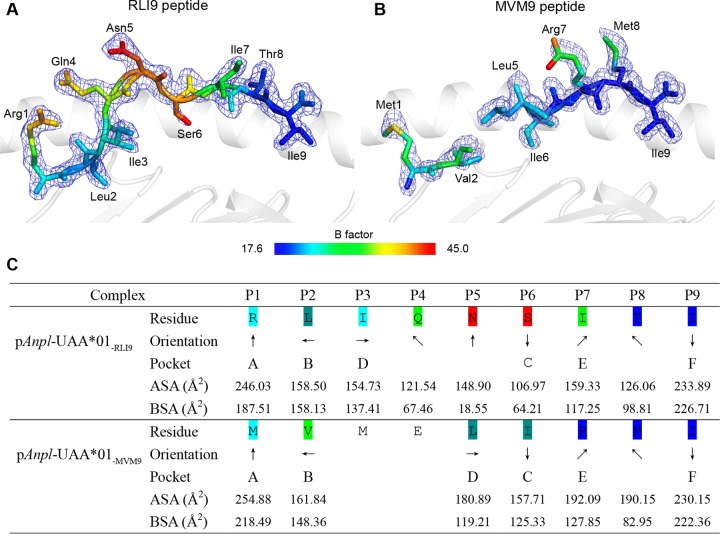
Divergent presentations of RLI9/MVM9 peptides presented by *Anpl*-UAA*01. (A and B) Electron densities and overall conformations of the structurally defined peptides RLI9 and MVM9. Simulated annealing omit maps (CNS) calculated for the two peptides are shown in blue at a contour of 1.5, and the coloration of the two peptides is according to the isotropic B factors. (C) General side chain orientations and the different interfacing areas of RLI9/MVM9 presented by *Anpl*-UAA*01 PBG, as viewed in profile from the peptide N terminus toward the C terminus. Black arrows indicate the directions in which the residues point: up is toward the T cell receptor, down is toward the floor of the peptide binding groove, left is toward the α1 helix domain, and right is toward the α2 helix domain. Pockets accommodating each residue are listed under the corresponding anchors within the peptide binding groove. ASA, accessible surface area of each residue; BSA, buried surface areas of the residues.

The conformations of IAV-MVM9 and IAV-RLI9 presented by *Anpl*-UAA*01 are distinct, especially in the middle region from the residue at position 3 (P3) to that at P6 (Fig. 5). The IAV-RLI9 peptide is clear on the electronic density map and adopts an “M” overall conformation, which is common to other pMHC I structures. The B factors of the IAV-RLI9 middle part are relatively higher than at the N and C termini, indicating that P4 to P6 are more flexible than other residues (Fig. 5A). In IAV-MVM9, the electronic density map is missing at P3 and P4, which means that these two residues are quite flexible (Fig. 5B). The side chain orientations of P5 are obviously different between the IAV-MVM9 and IAV-RLI9 peptides. In IAV-MVM9, the side chain of P5 stretches to the α2 helix but in RLI9, its side chain stretches upward and most parts are exposed from the PBG (Fig. 5C).

**FIG 6:**
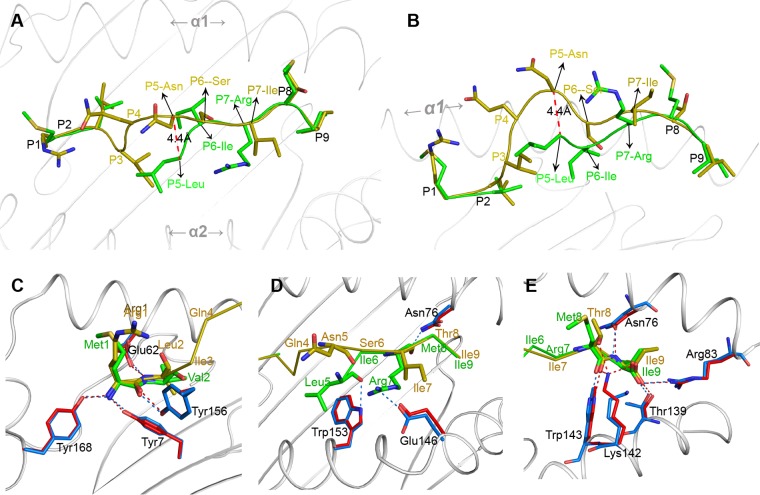
Comparisons of dissimilar conformations and interactions of RLI9/MVM9 peptides. (A and B) Superposition of RLI9 (olive) and MVM9 (green) presented by *Anpl*-UAA*01. To conveniently observe the different conformations of the two peptides, the side chains of residues are presented. Both the side (A) and top (B) views of the peptide alignments demonstrate that most conformational distinctions are located in the central region of the peptides, with the farthest distance between the backbone carbon atoms on P4 of the two peptides marked using red dashes. (C to E) Hydrogen bond comparisons of RLI9 and MVM9 peptides interacting with their PBGs. The interacting residues on PBG are shown (p*Anpl*-UAA*01–RLI9, red; p*Anpl*-UAA*01–MVM9, blue), and the hydrogen bonds are represented as dashed lines with the corresponding colors. (C) Similar interactions at the P1 and P2 positions of RLI9/MVM9 peptides with the residues in the A pocket of *Anpl*-UAA*01. (D) Different conformations and hydrogen bonds between the central region of peptides and *Anpl*-UAA*01. Three hydrogen bonds are formed in p*Anpl*-UAA*01–MVM9, and no hydrogen bonds are found in p*Anpl*-UAA*01–RLI9. (E) Interactions between the C-terminal residues of the RLI9/MVM9 peptides and their PBGs. Three more hydrogen bonds of RLI9 with the Lys^142^ residues of *Anpl*-UAA*01 are observed.

The conformations of the MVM9 and RLI9 peptides were significantly distinct when the two p*Anpl*-UAA*01 structures were superimposed (Fig. 6A and B); their deviation is focused mainly at the center, where the distance between the Cα atoms of MVM9 and RLI9 P5 residues can reach 4.4 Å. The N and C termini of the two peptides matched well. Structural analysis showed that the N termini of the MVM9 and RLI9 peptides (P1 and P2 residues) form the same hydrogen bonds with the PBG (Fig. 6C). From the P3 to P7 residues, there are no hydrogen bonds between RLI9 and the PBG. Although the P3 and P4 residues are missing in MVM9, three hydrogen bonds were found between P5, P7, and the PBG (Fig. 6D). These distinct peptide conformations of this part should be related to the differences in hydrogen bonds found here. In the C termini, the hydrogen bonds between the main chain of peptides and the PBG are the same; the only difference is that the Lys^142^ of p*Anpl*-UAA*01 forms 3 hydrogen bonds with the side chains of the P8 and P9 residues in the RLI9 peptide but does not form a bond with the MVM9 peptide (Fig. 6E).

These data suggest that the conformation of the middle section of the peptide presented by *Anpl*-UAA*01 is flexible and is determined by the peptide's specific amino acid sequence.

### *Anpl*-UAA*01 selects peptides by relying on the B and F pockets.

**FIG 7:**
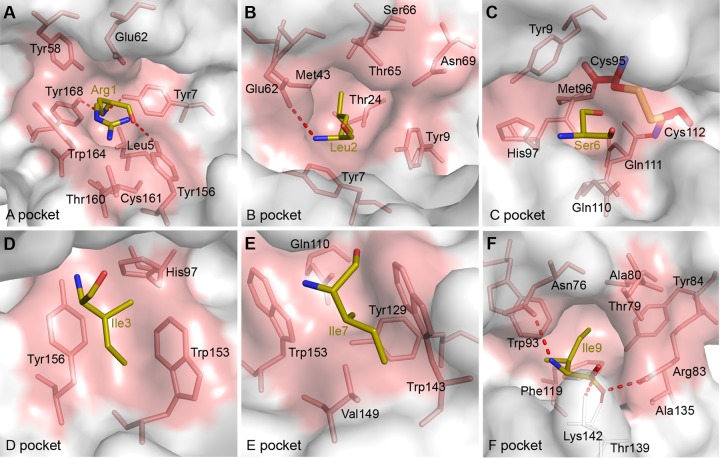
Composition of the pockets of *Anpl*-UAA*01. Pockets are shown as surface representations in light pink. Residues comprising these pockets (light pink) and the bound peptide RLI9 (C, olive; N, blue; O, red) are labeled. The hydrogen bonds between RLI9 and pockets are shown as red dashed lines. (A) Pocket A with the P1-Arg. (B) Pocket B with the P2-Leu. (C) Pocket C with the P6-Ser. The additional disulfide bond are shown as thick sticks and colored by atom type (C, red; N, blue; O, red; S, yellow). (D) Pocket D with the P3-Ile. (E) Pocket E with the P7-Ile. (F) Pocket F with the P_C_-Ile.

The compositions of the six pockets of *Anpl*-UAA*01 are shown in Fig. 7, and the interactions between peptides and these pockets are listed in Table 2. The RLI9 peptide was selected to illustrate the interactions with the six pockets in Fig. 6 because of its completeness.

**TABLE 2:** Interactions between the RLI9/MVM9 peptide and *Anpl*-UAA*01

Complex	Hydrogen bonds and salt bridges				van der Waals contact residues^*a*^
	Peptide		Heavy chain		
	Residue	Atom	Residue	Atom	
p*Anpl*-UAA*01–RLI9	P1-Arg	N	Tyr168	OH	Leu5, Tyr7, Tyr58, Glu62, Tyr156, Thr160, Trp164, Tyr168 (59)
		N	Tyr7	OH	
		O	Tyr156	OH	
	P2-Leu	N	Glu62	OE1	Tyr7, Tyr9, Thr24, Met43, Glu62, Thr65, Ser66, Tyr156 (46)
	P3-Ile				Thr65, His97, Trp153, Tyr156 (42)
	P4-Gln				Arg61, Thr65 (18)
	P5-Asn				(0)
	P6-Ser				Asn69, Ile72 (5)
	P7-Ile				Trp143, Lys142, Glu146, Val149, Ile72 (21)
	P8-Thr	OG1	Lys142	NZ	Ile72, Lys142, Asn76, Val75, Trp143 (31)
		O	Lys142	NZ	
		O	Trp143	NE1	
	P9-Ile	N	Asn76	OD1	Lys142, Trp143, Asn76, Thr139, Phe119, Thr79, Ala80, Arg83, Trp93 (69)
		OXT	Arg83	NH2 (S)	
		OXT	Thr139	OG1	
		O	Lys142	NZ (S)	
Total	11				291
p*Anpl*-UAA*01–MVM9	P1-Met	N	Tyr7	OH	Leu5, Tyr7, Tyr58, Arg61, Glu62, Tyr156, Trp164, Tyr168 (56)
		O	Tyr156	OH	
		N	Tyr168	OH	
	P2-Val	N	Glu62	OE1	Tyr7, Tyr9, Glu62, Thr65, Asn69, His97, Tyr156 (37)
	P5-Leu	O	Trp153	NE1	Gln152, Trp153, Tyr156 (33)
	P6-Ile				Tyr9, Asn69, Ile72, Phe73, Trp153 (26)
	P7-Arg	O	Asn76	ND2	Ile72, Asn76, Trp143, Glu146, Val149, Gln152 (31)
		NH2	Glu146	OE2 (S)	
	P8-Met	O	Trp143	NE1	Ile72, Val75, Asn76, Lys142, Trp143 (22)
	P9-Ile	N	Asn76	OD1	Asn76, Thr79, Ala80, Arg83, Trp93, Phe119, Thr139, Lys142, Trp143 (70)
		OXT	Arg83	NH2 (S)	
		OXT	Thr139	OG1	
Total	11				275

a Numbers in parentheses are the amounts of van der Waals force.

The A pocket of *Anpl*-UAA*01, composed of Leu^5^, Tyr^7^, Tyr^58^, Glu^62^, Tyr^156^, Thr^160^, Cys^161^, Trp^164^, and Tyr^168^, fixes P1-Arg by hydrogen bonds and strong van der Waals forces (VDWs) (Fig. 7A; Table 2). As in the known pMHC I structures, P1-Arg forms the hydrogen bonds with the A pocket by its main-chain atoms, and its side chain stretches upward out of the A pocket (45, 46). Therefore, although the binding between the A pocket and P1 residue is strong, the A pocket dose not play a restrictive role in the PBG of *Anpl*-UAA*01.

The B pocket is a primary anchor site and plays a restrictive role in peptide binding. The B pocket of *Anpl*-UAA*01 accommodates P2-Leu (Fig. 7B) The charged Glu^62^, which is on the top of the B pocket, can form a hydrogen bond with the main chain of P2-Leu. The side chain of the P2 residue inserts into the B pocket and is fixed by the VDWs provided by the surrounding residues (Table 2).

The C, D, and E pockets usually connect with residues in the middle part of the binding peptides. The amino acid compositions of these three pockets of *Anpl*-UAA*01 are shown in Fig. 7C to E. The C and D pockets can interact with the side chains of the P3 and P6 residues, respectively, but the E pocket can only interact with the main chain of the P7 residue, as the orientation of the P7 side chain faces upward (Table 2).

**FIG 8:**
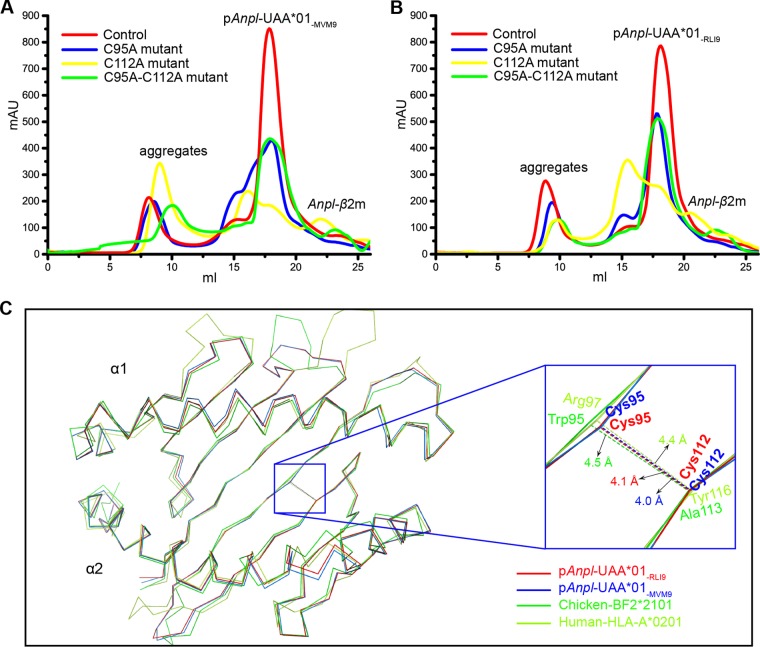
Mutants of *Anpl*-UAA*01 influence the *in vitro* refolding efficiency with L chain and RLI9/MVM9 peptides. The refolding products were analyzed by chromatography on a Superdex 200 10/300 GL column (GE Healthcare). Curves in different colors indicate different H chains. (A) Refolding results for the wild-type *Anpl*-UAA*01 and three mutants with the MVM9 peptide. (B) Refolding results with the RLI9 peptide. (C) The bottom β sheet of *Anpl*-UAA*01 is more compact than those in B21 and HLA-A2. All the structures are in ribbon models. The distances between C95 and C112 in *Anpl*-UAA*01 and their corresponding positions in B21 and HLA-A2 are labeled by dashed lines.

The additional disulfide bond formed by Cys^95^ and Cys^112^ is in the C pocket of *Anpl*-UAA*01. The impact of this disulfide bond on peptide binding was checked by the refolding of MVM9 and RLI9 peptides with the C95A, C112A, and C95A-C112A double mutant heavy chains. We found that that the mutant H chains can still form a complex with peptides, but the refolding is worse than that of the wild-type H chain, especially for the C112A mutant H chain (Fig. 8A and B). The reduced refolding efficiencies of the mutants indicated that the disulfide bound in the C pocket helps with the peptide binding of *Anpl*-UAA*01; however, the distances between the peptide residues and disulfide bond are over 5.0 Å, which means that their direct interactions are negligible (Fig. 6C). Previous studies have suggested that Cys^95^ and Cys^112^ do not meet the preferred geometry to form the disulfide bond (30), and our p*Anpl*-UAA*01 structures show that the distance between Cys^95^ and Cys^112^ is less than that for the corresponding positions in HLA-A2 and B21 (Fig. 8C). To some extent, the additional disulfide bond of *Anpl*-UAA*01 alters the bottom of the C pocket and strengthens the stability. Improved peptide binding efficiency may strengthen the stability of the C pocket with the additional disulfide bound.

The F pocket is the most important anchor site at the C terminus of PBG. The F pocket of *Anpl*-UAA*01 is composed of Asn^76^, Thr^79^, Ala^80^, Arg^83^, Tyr^84^, Trp^93^, Phe^119^, and Ala^135^, and it has a strong binding affinity with P9-I (Fig. 7F). The main chain of P9-I can form 2 salt bridges with Arg^83^ and Lys^142^ and 2 hydrogen bonds with Asn^76^ and Thr^139^ in the F pocket. The side chain of P9-I inserts into the F pocket and is fixed by the strong hydrophobic forces (Table 2).

**FIG 9:**
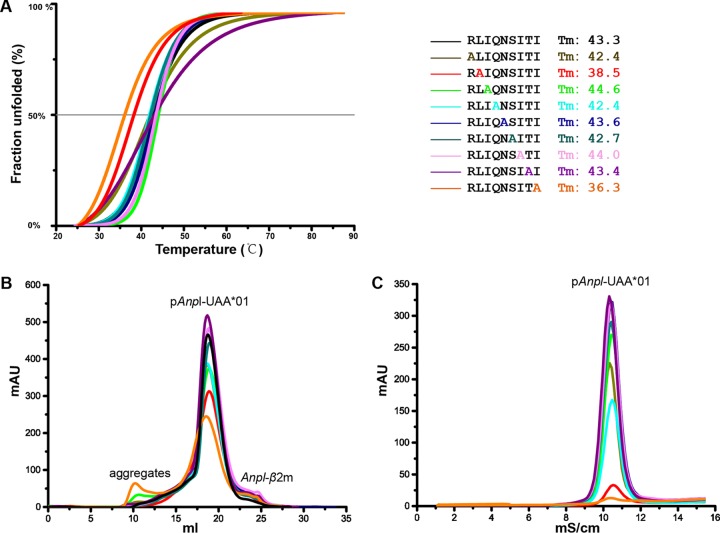
P2 and P9 of the peptide are critical for binding to *Anpl*-UAA*01. The denaturation and elution curves of the complexes with the different peptides are indicated in different colors. (A) Thermostabilities of *Anpl*-UAA*01 with the RLI9 peptide and its substitutions (residues at each position in RLI9 are replaced with alanine). Circular dichroism spectropolarimetry was utilized to assess the thermostabilities of purified p*Anpl*-UAA*01 complexes. Denaturation was monitored at 218 nm as the temperature was ramped up from 25 to 75°C at 1°C/min. Shown here are the data for fitting to the denaturation curves using the Origin 9.1 program (OriginLab).The *T_m_*s of different peptides are indicated by the gray line at 50% fraction unfolded. (B and C) Peptide-induced assembly and stabilization assay of *Anpl*-UAA*01 and *Anpl*-β2m with RLI9 and its mutants by *in vitro* refolding. (B) Gel filtration chromatograms of the refolded products obtained using a Superdex 200 10/300 GL column (GE Healthcare). The aggregated H chain, the correctly refolded p*Anpl*-UAA*01 complex (∼45 kDa), and the extra β2m are indicated. The refolding efficiencies are represented by the heights of p*Anpl*-UAA*01 complex peaks. A higher peak indicates a better efficiency of the peptide to help the MHC renature. (C) Results of further stabilization assays of the refolded products tested by anion exchange. A higher peak here also indicates better stability. With peptides P2-A and P9-A, the refolded complex proteins dissociated at the corresponding eluting NaCl concentration (9% to 11%), implying poor stabilities.

In order to determine the primary anchor residues of *Anpl*-UAA*01-presenting peptides and the vital restriction pockets for peptide binding, the RLI9 peptide was mutated by alanine scanning and circular dichroism (CD) spectral analysis was used to test the stabilities of *Anpl*-UAA*01 complexes with these mutant peptides (Fig. 9). The wild-type RLI9 peptide was used as a control, and its midpoint transition temperature (*T_m_*) value was 43.3°C. Among all of the alanine mutant peptides, only the *T_m_* values of the P2-Ala and P9-Ala mutant peptides were significantly lower than that of the wild-type RLI9 peptide, indicating that the side chains of P2 and P9 play key roles in RLI9 peptide binding and that these two residues are the primary anchor residues. The pockets (B and F) at the two termini of the *Anpl*-UAA*01 PBG anchor the peptides and determine the peptide binding motif of *Anpl*-UAA*01.

### The peptide binding motif of *Anpl*-UAA*01 is most similar to that of HLA-A2.

**FIG 10:**
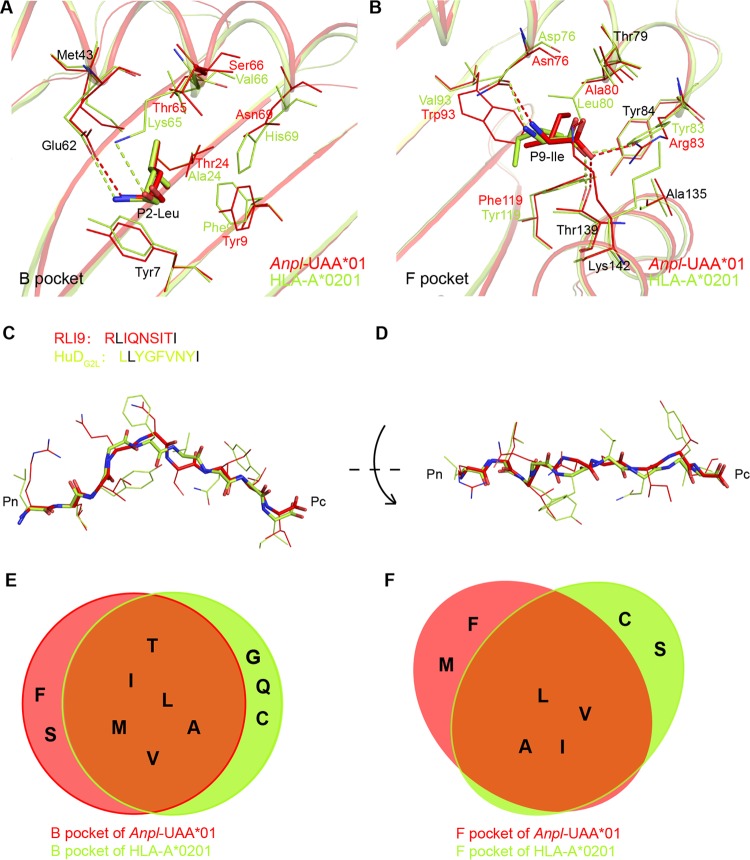
Alignments of B pocket, F pocket, and binding peptides indicate similar peptide binding motifs of *Anpl*-UAA*01 with HLA-A*0201 (PDB code 3PWN). Residues making up these pockets and binding peptides are as follows: for p*Anpl*-UAA*01, C, red; N, blue; O, red; and for pHLA-A*0201, C, lemon; N, blue; O, red). (A and B) B and F pocket alignments of *Anpl*-UAA*01 with HLA-A*0201. The same compositions of P2-Leu and P9-Ile in both RLI9/HuD_G2L_ peptides are shown using the same color as their H chains. Hydrogen bonds formed by P2/P9 and their H chains are represented as dashed lines with each corresponding color. (C and D) Side and top views of the RLI9 peptide alignment with HuD_G2L_ peptide presented by HLA-A*02. Both of the peptides' main chains are shown as bold sticks. Shared P2-Leu and P9-Ile in RLI9/HuDG2L peptides are colored black in their sequences in the upper left corner. (E and F) Residues fitting the B and F pockets of *Anpl*-UAA*01 and HLA-A*0201 can mostly overlap. Residues within the solid red circle are motifs for *Anpl*-UAA*01, and those within the circle are motifs for HLA-A*0201.

The peptide binding manner of p*Anpl*-UAA*01 is similar to those of some mammalian pMHCs but not similar to that of chicken pMHC I; only the B and F pockets are the key anchor caves, similar to what is observed in human MHC I (also named human lymphocyte antigen [HLA]). By comparison with the solved structures in the Protein Data Bank (PDB), the peptide conformation and binding manner of p*Anpl*-UAA*01 were found to be most similar to those of HLA-A2 (Fig. 10). In the B pockets of *Anpl*-UAA*01 and HLA-A2, the side chains of P2-Leu residues have similar orientations and are fixed in a similar way by the residues at the same positions in the two pockets (Fig. 10A). A similar situation was also found in the two F pockets, where the hydrogen bonds fixing P9-I are almost the same (Fig. 10B). The peptide presentations and pocket compositions make the peptide conformations of p*Anpl*-UAA*01 and pHLA-A2 almost identical (Fig. 10C and D).

Previous studies have shown that the B pocket of HLA-A2 prefers hydrophobic anchor residues, such as I, L, V, A, and F, and can also accommodate the same polar residues, such as T. The most frequently occurring anchor residues in the F pocket of HLA-A2 are I, V, and L. The two peptides presented by *Anpl*-UAA*01, MVM9 and RLI9, perfectly fit the peptide binding motif of HLA-A2. Eighteen additional peptides from IAV and 11 mutant peptides were used to verify the peptide binding motif of *Anpl*-UAA*01 (Table 3). The results confirmed that the peptide binding motifs of these two MHC I molecules from different species are mostly overlapping (Fig. 10E and F). In brief, the nonapeptide binding motif of *Anpl*-UAA*01 is x-(A, V, I, L, M, T, S, or F)-x-x-x-x-x-x-(A, V, I, L, M, or F).

**TABLE 3:** Predicted peptides used in this study and their binding to *Anpl*-MHC I, evaluated by *in vitro* refolding

Name	Derived protein	Position	Sequence	% random^*a*^	Stability with *Anpl*-MHC I^*b*^
IAY9	Nucleocapsid protein (IAV)	45–53	IAYERMCNI	0.185	++
RTS9	Nucleocapsid protein (IAV)	22–30	RTSDMRTEI	0.168	++
MVM9	Nucleocapsid protein (IAV)	17–25	MVMELIRMI	0.172	++
RLI9	Nucleocapsid protein (IAV)	55–63	RLIQNSITI	0.245	++
WMA9	Nucleocapsid protein (IAV)	68–76	WMACHSAAF	0.213	++
IFL9	Nucleocapsid protein (IAV)	18–26	IFLARSALI	0.125	++
VSG9	Nucleocapsid protein (IAV)	28–36	VSGIGRFYI	0.134	++
ATA9	Nucleocapsid protein (IAV)	53–61	ATAGLTHLM	0.200	+/−
SLAP5	Hemagglutinin (IAV)	25–33	TSADQQSLY	0.100	+/−
SLAP9	Polymerase PA (IAV)	6–14	GTFDLGGLY	0.041	+/−
AI-4	Hemagglutinin (IAV)	442–450	VAMENQHTI	0.387	++
EN5	Gag protein (IAV)	226–234	MTARFIRGL	0.130	++
KMN-P9I	Hemagglutinin (IAV)	402–410	KMNTQFIAI	0.207	++
NS1-VKN	Nonstructural protein 1 (IAV)	174–182	VKNAVGVLI	0.054	+/−
PA-ENK	Polymerase PA (IAV)	613–621	ENKSETWPI	0.035	+/−
PA-KTN	Polymerase PA (IAV)	497–505	KTNLYGFII	0.150	++
HA-ITN	Hemagglutinin (IAV)	393–401	ITNKVNSVI	0.162	++
HA-YIN	Hemagglutinin (IAV)	182–190	YINDKGKEV	0.073	+/−
PA-RRN	Polymerase PA (IAV)	442–450	RRNYFTAEV	0.059	+/−
PB1-KM	Polymerase PB1 (IAV)	531–539	KNNMINNDL	0.046	+/−
RLI9-P1A	Mutant RLI9		ALIQNSITI		++
RLI9-P2A	Mutant RLI9		RAIQNSITI		+/−
RLI9-P3A	Mutant RLI9		RLAQNSITI		++
RLI9-P4A	Mutant RLI9		RLIANSITI		++
RLI9-P5A	Mutant RLI9		RLIQASITI		++
RLI9-P6A	Mutant RLI9		RLIQNAITI		++
RLI9-P7A	Mutant RLI9		RLIQNSATI		++
RLI9-P8A	Mutant RLI9		RLIQNSIAI		++
RLI9-P9A	Mutant RLI9		RLIQNSITA		+/−
RLI9-P9V	Mutant RLI9		RLIQNSITV		+/−
RLI9-P9L	Mutant RLI9		RLIQNSITL		++

a Base value for estimating the binding affinities of peptides with the NetMHCpan 3.0 server (http://www.cbs.dtu.dk/services/NetMHCpan/): rank threshold for strongly binding peptides, 0.100; rank threshold for weakly binding peptides, 1.000.

b ++, peptide binds strongly and can tolerate anion-exchange chromatography; +/−, peptide binds *Anpl*-MHC I but cannot tolerate anion-exchange chromatography.

### Many IAV peptides match the motif of p*Anpl*-UAA*01.

**FIG 11:**
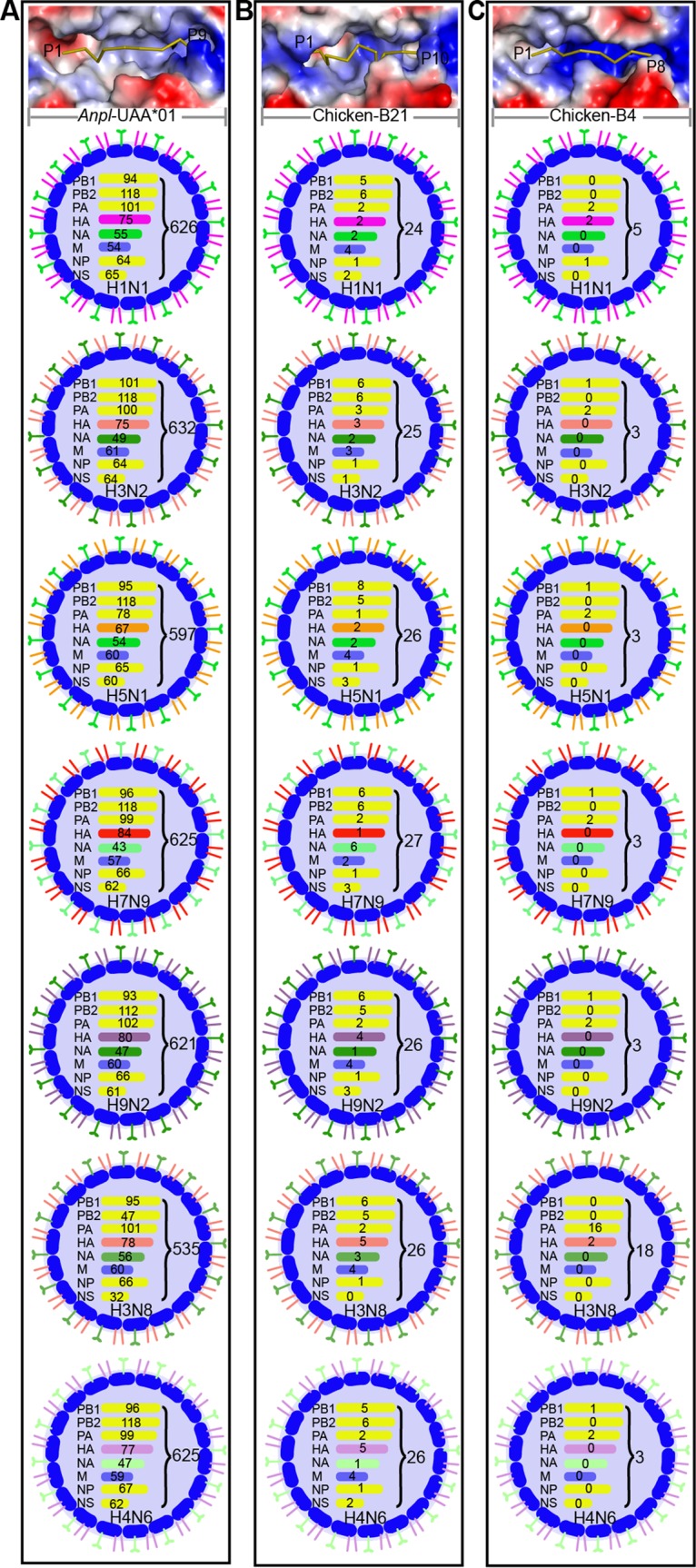
Peptide predictions from different influenza virus subtypes according to the distinct binding motifs of *Anpl*-UAA*01, BF2*0401, and BF2*2101. Genome-wide scanning results of peptides matching the motifs of *Anpl*-UAA*01 (A), BF2*0401 (B), and BF2*2101 (C) are as follows: *Anpl*-UAA*01, x-(A, V, I, L, M, T, S, or F)-x-x-x-x-x-x-(A, V, I, L, M, or F); BF2*0401: x-(D, E)-x-x-(D, E)-x-x-E; BF2*2101, x-(H, K, or R)-x-x-x-x-x-(E or D)-x-(A V, L, I, F, or W), x-(E or D)-x-x-x-x-x-L-x-(A, V, L, I, F, or W). The numbers of peptides derived from the PB1, PB2, PA, HA, NA, M, NP, and NS proteins of each IAV subtype are marked, as well as the total numbers. Vacuum electrostatic surface potential presentations (red, negative; blue, positive; gray, neutral) and predominant lengths of binding peptides of PBGs in *Anpl*-UAA*01, BF2*0401, and BF2*2101 are shown below. The peptides are shown as ribbons (olive) with their lengths labeled: nonamers for *Anpl*-UAA*01, octamers for B4, and 10-mers for B21.

Using the motif of *Anpl*-UAA*01 that we identified, the proteomes of several IAV strains were screened to identify the epitope peptides that could be presented by *Anpl*-UAA*01, including the H1N1, H3N2, H3N8, H4N6, H5N1, H7N9, and H9N2 subtypes (47–53). There were approximately 600 candidate nonapeptides from different IAV strains matching the peptide binding motif of *Anpl*-UAA*01 (Fig. 11). Most selected peptides also fit the motif of HLA-A2, and 11 nonapeptides were proven to activate HLA-A2-restricted CTL responses efficiently according to IEDB data (http://www.iedb.org/) (54). The *in vitro* refolding results for 31 peptides confirmed that the predicted IAV epitopes could be bound by *Anpl*-UAA*01 (Table 3).

Based on the motifs of chicken B4 and B21, the longer peptides from IAV strains were also screened. There are fewer than 20 octapeptides for B4 and 24 to 27 decapeptides for B21 (Fig. 11), which are much less than the numbers of peptides found in *Anpl*-UAA*01.

## DISCUSSION

The most distinguishable feature of *Anpl*-UAA*01 was the additional disulfide bond in the PBG. The mutant experiments showed that this disulfide bond located in the C pocket can increase the efficiency of peptide binding to p*Anpl*-UAA*01. Although all of the mutants could still form complexes with the RLI9/MVM9 peptide and β2m, their refolding efficiencies were significantly lower than that of the wild type (Fig. 8A and B). Without the peptide, this disulfide bond did not increase the refolding production of the complex of *Anpl*-UAA*01 and β2m (Fig. 3). These data indicate that this additional disulfide bond can improve peptide binding to *Anpl*-UAA*01 and lead to the increased formation of a trimer complex; however, no significant contacts between the presented peptide and disulfide bond were identified. We also discovered that the additional disulfide bond makes the bottom β sheet of the C pocket of *Anpl*-UAA*01 more compact than in other MHC I molecules. The refolding effect of the C112A mutant indicated that the free side chain of Cys^95^ could disturb peptide binding in the C pocket region (Fig. 8A and B). Therefore, we hypothesize that this additional disulfide bond provides a more stable C pocket, which helps with peptide presentation of *Anpl*-UAA*01.

Another characteristic of the *Anpl*-UAA*01 structure is the intensive interaction between its H and L chains. In comparison with other known pMHC I structures, p*Anpl*-UAA*01 has the greatest interface area and many interchain bonds (Fig. 5). The interface area in ducks and chickens is significantly larger than that in mammals. Compared with BF2*2101 (32), p*Anpl*-UAA*01 had a 100-Å^2^-larger interface area and 4 more hydrogen bonds; this relatively stronger interaction could lead to the formation of complexes containing only *Anpl*-UAA*01 and β2m through *in vitro* refolding, which has never been identified in pMHC I structures of other species (33, 39–41).

*Anpl*-UAA*01 is similar to human HLA in peptide presentation (but not to chicken BF2), although the amino acid identities among *Anpl*-UAA*01 and BF2 molecules are much higher than those between *Anpl*-UAA*01 and HLA (>60% versus <50%, respectively). There are only two primary anchor pockets in p*Anpl*-UAA*01, the B and F pockets, which is common in HLA molecules (Fig. 10A and B). Using structural analysis and mutant peptide refolding tests, we found that the peptide binding motif of p*Anpl*-UAA*01 overlaps with HLA-A*0201 to a large degree. The B pocket of *Anpl*-UAA*01 can accommodate extensive uncharged residues, and the F pocket can accommodate multiple hydrophobic residues (Fig. 10E). However, BF2 exhibits peptide-presenting strategies that differ from those of HLA. B4 has a narrow and highly charged PBG, which limits the binding peptides that must fit its B, C, and F pockets together (33). B21 has a large central cavity and flexible Arg9, which make its binding motif promiscuous with three anchor residues (32). The moderate limits of the peptide binding motif suggest that ducks can accommodate more peptides from pathogens than chickens.

The role of MHC I mediating the CTL response in clearance of IAV has been confirmed in human and mouse. Although substantial data on anti-IAV CTL responses in duck and chicken are scarce, some evidence indicates that duck MHC genes respond to infection with IAV (13, 55). The studies showed that duck MHC locus genes (MHC I/II and TAP) are overexpressed after infection with IAV, especially the duck MHC I gene, and under the condition of HP-IAV infection its expression could be increased by about 1,000 times in the lung (13). Unlike mammals, both ducks and chickens have a “minimal MHC” region with a limited set of genes and express only one dominant MHC I allele. The “minimal MHC” in chickens is critical to defense against a particular pathogen because it is completely dependent on whether or not it can load peptides from that pathogen. The best illustrated example is that chickens of the BF2*2101 genotype could defend against MDV, while chickens of the BF2*0401 genotype could not (32, 33, 56). The difference in duck versus chicken responses to IAV might also relate to the different peptide loading abilities of duck and chicken MHC I. Approximately 600 nonapeptides from different IAV strains matching the motif of p*Anpl*-UAA*01 were identified in this study, which is much higher than the numbers of BF2*2101 and BF2*0401 from chicken (Fig. 11; see Data Set S1 in the supplemental material). Therefore, the duck *Anpl*-UAA*01 should exhibit stronger resistance to IAV strains than chicken BF2*2101 and BF2*0401 (57, 58). In addition, the *Anpl*-UAA*01 structure showed that it can present IAV peptides in distinct conformations, which is believed to be useful for activating the T cell repertoire and inducing stronger CTL responses. Our study illustrates the structural basis of duck MHC I and provides a novel approach for explaining why ducks are more resistant to HP-IAV.

## MATERIALS AND METHODS

### Prediction and synthesis of IAV-derived peptides.

All nonamer peptides that potentially bound to *Anpl*-UAA*01 were predicted using the NetMHCpan 3.0 server (http://www.cbs.dtu.dk/services/NetMHCpan/) (59, 60). The 31 peptides used in this study (Table 3) were synthesized and purified to 90% by reverse-phase high-performance liquid chromatography (HPLC) and mass spectrometry (SciLight Biotechnology). These peptides were stored in lyophilized aliquots at −80°C after synthesis and dissolved in dimethyl sulfoxide (DMSO) before use.

### Protein preparation.

DNA fragments encoding extracellular domains of *Anpl*-UAA*01 (GenBank accession no. AB115245, residues 1 to 270 of the mature protein with EcoRI and HindIII restriction sites) (37) and *Anpl*-β2-microglobulin (*Anpl*-β2m) (GenBank accession no. AB246408, residues 1 to 101 of the mature protein) were synthesized by Shanghai Invitrogen Life Technologies and then cloned into pET21a(+) vectors (Novagen) and expressed in Escherichia coli BL21(DE3). The recombinant *Anpl*-UAA*01 and β2m were expressed in inclusion bodies and purified as previously described (61, 62). Finally, the *Anpl*-UAA*01 heavy chain and *Anpl*-β2m bodies were separately dissolved in 6 M guanidinium chloride buffer to a protein concentration of 30 mg/ml.

### Assembly of the p*Anpl*-UAA*01 complex.

To assemble the p*Anpl*-UAA*01 complex, the peptide, *Anpl*-UAA*01, and *Anpl*-β2m inclusion bodies were refolded (in a 1:1:1 molar ratio) according to the gradual dilution method that we described previously (39, 63). After a 24-h refolding step at 277 K, the remaining soluble portion of the complex was concentrated and purified using a Superdex 200 16/60 column (GE Healthcare), followed by Resource Q anion-exchange chromatography (GE Healthcare). Purified proteins were buffer exchanged with 10 mM Tris-HCl and 50 mM NaCl at a pH of 8.0.

### Crystallization and data collection.

The purified p*Anpl*-UAA*01 complex was ultimately concentrated to 10 mg/ml. After mixing with reservoir buffer at a 1:1 ratio, the purified protein was crystallized using the sitting-drop vapor diffusion technique at 277 K. Index, Crystal Screen I/II, and Crystal Screen Cryo I/II kits (Hampton Research, Riverside, CA) were used to screen for optimal crystal growth conditions. After several days, crystals (p*Anpl*-UAA*01–MVM9 and p*Anpl*-UAA*01–RLI9) were observed with solutions NO.7 from the Crystal Screen Cryo II kit (8% polyethylene glycol 1000, 8% [wt/vol] polyethylene glycol 8000, and 20% [vol/vol] glycerol) and NO.43 from the Crystal Screen Cryo I kit (24% [wt/vol] polyethylene glycol 1500 and 20% [vol/vol] glycerol), respectively. Diffraction data for p*Anpl*-UAA*01 crystals were collected to resolutions of 1.71 Å (p*Anpl*-UAA*01–MVM9) and 2.06 Å (p*Anpl*-UAA*01–RLI9) at the Shanghai Synchrotron Radiation Facility (SSRF) using beamline BL17U at a wavelength of 1.5418 Å (Shanghai, China) (64).The crystals were first soaked in reservoir solution containing 25% glycerol as a cryoprotectant and were then flash-cooled in a stream of gaseous nitrogen at 100 K (65). The collected intensities were indexed, integrated, corrected for absorption, scaled, and merged using the HKL2000 package (66).

### Structure determination and refinement.

The crystals of p*Anpl*-UAA*01–MVM9/RLI9 all belong to the P1211 space group, and their structures were solved by molecular replacement using Molrep and Phaser in the CCP4 package, with the chicken BF2*0401 structure (PDB code 4E0R) as the search model (67–69). Extensive model building was performed by hand with COOT (70), and restrained refinement was performed using REFMAC5. Additional rounds of refinement were performed using the Phenix refine program implemented in the PHENIX package (71) together with isotropic atomic displacement parameter (ADP) refinement and bulk solvent modeling. The stereochemical quality of the final model was assessed with the PROCHECK program (72). Detailed information about collection and refinement is shown in Table 1.

### Structural analysis and generation of illustrations.

Peptide-contacting residues were identified using the program CONTACT and were defined as residues containing an atom within 3.3 Å of the target partner (69). Structural illustrations and electron density-related figures were generated using the PyMOL molecular graphics system (http://www.pymol.org/). Solvent-accessible surface areas and the B factor were calculated with CCP4.

### Preparation of Cys-to-Ala mutants of p*Anpl* MHC I.

To investigate the function of the additional disulfide bond in *Anpl*-UAA*01, Cys^95^ and Cys^112^ were mutated to Ala by overlap PCR (the primers used for Cys^95^-to-Ala mutation were 5′-GGCAGGCGATGCATGGCTGTG-3′ and 5′-GCCATGCATCGCCTGCCATG-3′ and those for Cys^112^-to-Ala mutation were 5′-CAACAAGCGGGCTATGATGGG-3′ and 5′-CCCATCATAGCCCGCTTGTTG-3′, where the underlined sequences mutated the codon encoding Ala). These mutants were inserted into the pET21a vector and expressed in BL21(DE3) cells. The mutants were termed *Anpl*-UAA*01-C95A, *Anpl*-UAA*01-C112A, and *Anpl*-UAA*01-C95A-C112A, respectively. Recombinant mutants were expressed as inclusion bodies and further purified as described above. The mutant inclusion bodies were refolded with *Anpl*-β2m using the *in vitro* gradual dilution method as described above (61, 62). In addition, all of the mutant p*Anpl*-UAA*01 complexes formed by refolding were further purified by gel filtration and anion-exchange chromatography as described above (39, 63).

### CD spectra and thermal unfolding.

Circular dichroism (CD) experiments for the p*Anpl*-UAA*01 with parental RLI9 or mutant peptides were performed on a Jasco J-810 spectropolarimeter equipped with a water-circulating cell holder. The CD spectra were collected at a protein concentration of 8 mΜ in pH 8.0 Tris buffer (20 mM Tris and 50 mM NaCl), using a 1-mm-optical-path-length cuvette with ellipticity at 218 nm. Thermal denaturation curves were determined as the temperature was raised from 25 to 80°C at a linear rate of 1°C/min. The temperature of the sample solution was directly measured with a thermistor. The fraction of unfolded protein was calculated from the mean residue ellipticity (θ) using the standard method. The unfolded fraction (%) was expressed as (θ − θN)/(θU − θN), where θN and θU are the mean residue ellipticity values in the fully folded and fully unfolded states, respectively. The midpoint transition temperature (*T_m_*) was determined by fitting data to the denaturation curves using the Origin 9.1 program (OriginLab) (73).

### Accession number(s).

The coordinates and structure factors for p*Anpl*-UAA*01–RLI9 and p*Anpl*-UAA*01–MVM9 have been deposited in the Protein Data Bank (http://www.rcsb.org/pdb/home/home.do) under accession numbers 5GJX and 5GJY, respectively.

## Supplementary Material

Supplemental material
