# Supplementary material for: Structural Definition of Duck Major Histocompatibility Complex Class I Molecules That Might Explain Efficient Cytotoxic T Lymphocyte Immunity to Influenza A Virus
Source: J Virol. 2017 Jun 26;91(14):e02511-16. doi: 10.1128/JVI.02511-16 (PMC5487541; doi:10.1128/JVI.02511-16)
Supplement: Supplemental material [file supp_91_14_e02511-16__index.html]

Structural Definition of Duck Major Histocompatibility Complex Class I Molecules That Might Explain Efficient Cytotoxic T Lymphocyte Immunity to Influenza A Virus — Supplemental material 

# Structural Definition of Duck Major Histocompatibility Complex Class I Molecules That Might Explain Efficient Cytotoxic T Lymphocyte Immunity to Influenza A Virus

## Supplemental material

- Supplemental file 1 -

  Data Set S1 (Screening result of peptides in the proteomes of five IAV strains based on the motifs of Anpl-UAA\*01, BF2\*0401, and BF2\*2101.)

  XLSX, 148K
